# Pathophysiology-guided nutritional strategies for persistent inflammation, immunosuppression, and catabolism syndrome: an immunometabolic perspective

**DOI:** 10.3389/fimmu.2026.1968812

**Published:** 2026-10-20

**Authors:** Zhiyang Wu, Bo Yao

**Affiliations:** Department of Critical Care Medicine, Qilu Hospital (Qingdao), Cheeloo College of Medicine, Shandong University, Qingdao, Shandong, China

**Keywords:** anabolic resistance, chronic critical illness, circadian-metabolic regulation, immunometabolism, immunonutrition, nutritional therapy, persistent inflammation, immunosuppression, and catabolism syndrome (PICS), precision nutrition

## Abstract

Persistent inflammation, immunosuppression, and catabolism syndrome (PICS) is a phenotype of chronic critical illness characterized by unresolved inflammation, impaired immune competence, sustained catabolism, skeletal muscle loss, recurrent infections, and poor functional recovery. Although guideline-based nutritional support remains fundamental, persistent immunometabolic abnormalities may limit responsiveness to nutrient delivery and contribute to anabolic resistance, impaired substrate utilization, and defective tissue repair. This mini-review examines biological mechanisms limiting nutritional responsiveness in PICS and evaluates evidence for pathophysiology-guided nutritional strategies. A structured literature search of PubMed/MEDLINE, Embase, and Web of Science was conducted from database inception through September 6, 2026, prioritizing English-language clinical guidelines, clinical studies, and mechanistic or translational evidence relevant to PICS, critical illness, nutritional responsiveness, and immunometabolic nutrition. We propose a framework addressing major barriers to recovery through strategies to restore anabolic responsiveness with protein, leucine, and rehabilitation; modulate inflammatory resolution and immune dysfunction with omega-3 fatty acids and arginine; optimize nutrient timing and circadian-metabolic regulation; and maintain a metabolic milieu conducive to nutrient utilization. Because direct interventional evidence in well-defined PICS populations remains limited, these strategies should complement rather than replace guideline-based nutritional support. The framework is conceptual and hypothesis-generating, and prospective PICS-specific studies incorporating biological stratification and clinically meaningful recovery outcomes are needed to determine its translational value.

## 1 Introduction

Advances in intensive care have improved survival, but some survivors develop chronic critical illness (CCI) with persistent inflammation, immune dysfunction, metabolic disturbance, and functional decline (1, 2). Persistent inflammation, immunosuppression, and catabolism syndrome (PICS) is a CCI phenotype after infection, trauma, surgery, or other insults, characterized by unresolved inflammation, impaired immunity, and sustained catabolism (3, 4). It is generally recognized during prolonged critical illness, commonly after approximately 14 days, when these abnormalities coexist. Operational definitions incorporate persistent inflammatory activity, lymphopenia or other impaired immune competence, and catabolic features such as weight or skeletal muscle loss and impaired anabolic recovery; however, thresholds vary and no universally validated definition or biomarker panel exists. PICS-1 distinguishes this phenotype from post-intensive care syndrome (PICS-2); here, PICS refers to the former (5). Unlike normal recovery, PICS entails prolonged inflammation, impaired host defense, and failure to restore anabolic balance (3, 6). These operational features should be interpreted longitudinally because PICS develops over time and overlaps clinically with other causes of prolonged critical illness.

PICS reflects immunometabolic interactions. Persistent innate activation by pathogen- and damage-associated molecular patterns sustains IL-6 and TNF-α (7, 8), while prolonged activation impairs antigen presentation, lymphocyte function, and antimicrobial capacity (9). Concurrently, protein breakdown, mitochondrial dysfunction, impaired muscle protein synthesis, and anabolic resistance occur (10, 11), supporting PICS as an immunometabolic disorder (12).

Guideline-based nutrition with timely enteral nutrition and energy/protein provision remains fundamental (13, 14). In PICS, inflammation and altered nutrient metabolism may promote insulin resistance, abnormal substrate use, and suppressed anabolic pathways (10, 15). Persistence despite guideline-concordant delivery has been termed nutrition resistance (5, 16), such that adequacy alone may not overcome anabolic and metabolic dysfunction (15–17). Guidelines remain essential (13, 14) but derive from heterogeneous ICU populations. Nutritional strategies may therefore need to address immune regulation, metabolic adaptation, and tissue recovery (17). Requirements and responsiveness also vary across acute illness, stabilization, and ICU/post-ICU recovery; strategies should therefore be phase-specific rather than uniform (18).

This mini-review evaluates clinical, translational, and mechanistic evidence on nutritional responsiveness in PICS or prolonged critical illness and considers nutritional and related strategies targeting anabolic resistance, immune dysfunction, circadian–metabolic disruption, and metabolic instability. Building on previous reviews, we organize evidence by biological barriers to host responsiveness rather than individual nutrients. The framework identifies mechanistic links, evidence gaps, and research priorities and is conceptual and hypothesis-generating rather than a validated clinical algorithm. **Figure 1** depicts the proposed mechanistic relationships, candidate intervention nodes, and key areas of extrapolation requiring PICS-specific validation.

**Figure 1:**
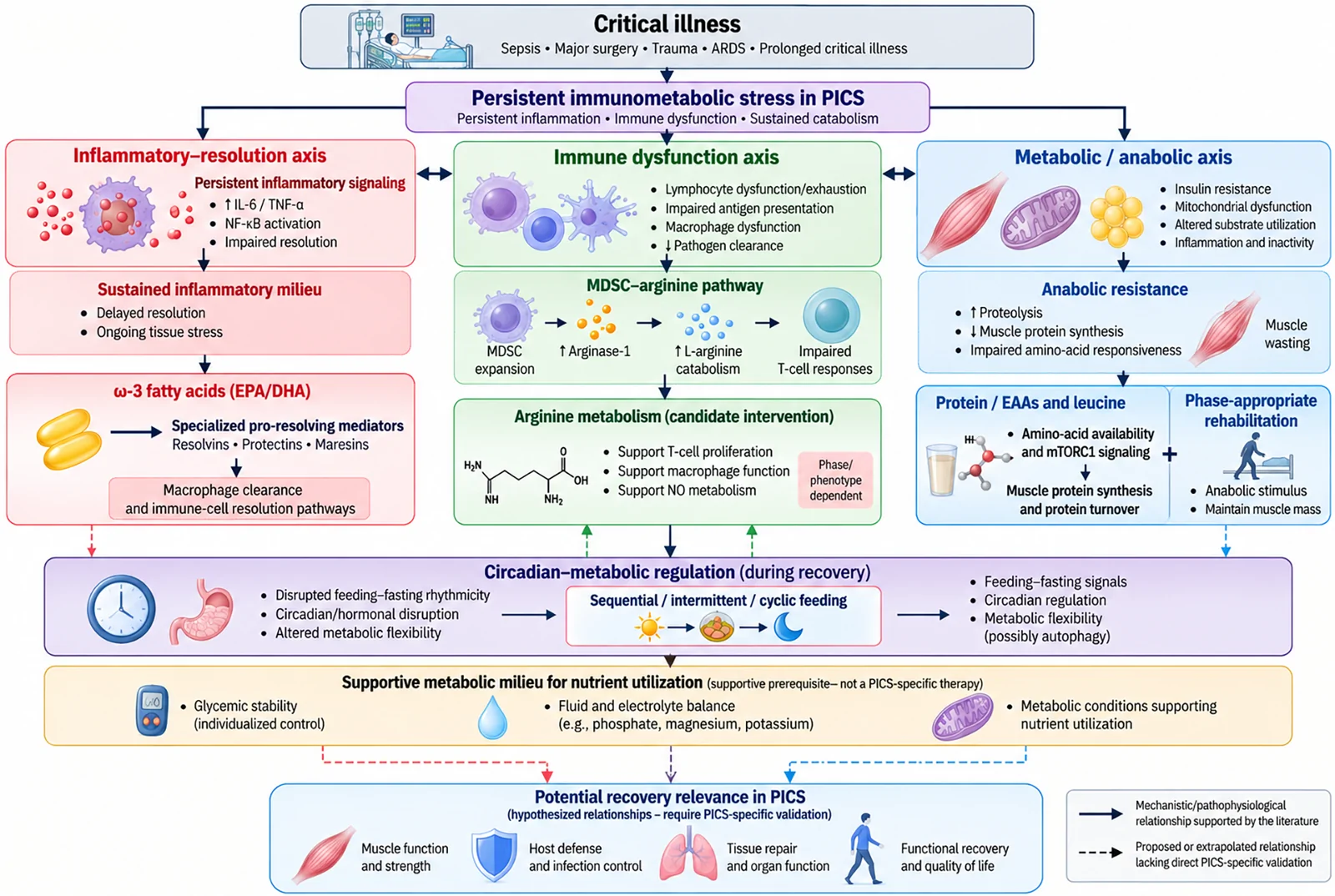
Mechanistic framework for pathophysiology-guided immunometabolic nutrition in PICS. Critical illness may lead to persistent immunometabolic stress characterized by interconnected inflammatory, immune, and metabolic/anabolic dysfunction. The framework depicts selected mechanistic pathways discussed in the text, including persistent inflammatory signaling and impaired resolution; the MDSC–arginase-1–L-arginine pathway and impaired T-cell responses; and inflammation-, inactivity-, and metabolic dysfunction-associated anabolic resistance, impaired amino-acid responsiveness, and muscle wasting. Candidate nutritional and rehabilitation approaches are positioned at the biological nodes they may influence. These include the EPA/DHA–specialized pro-resolving mediator pathway, arginine metabolism and immune-cell function, and protein/essential amino acids and leucine–mTORC1 signaling together with phase-appropriate rehabilitation. Circadian–metabolic regulation and phase-adapted feeding patterns are shown as an additional recovery-related pathway, whereas glycemic and fluid/electrolyte management are positioned as components of a supportive metabolic milieu for nutrient utilization rather than stand-alone PICS-specific therapies. Solid arrows indicate mechanistic or pathophysiological relationships supported by the literature discussed in this review; dashed arrows indicate proposed or extrapolated relationships lacking direct PICS-specific validation. Downstream recovery outcomes are therefore presented as potential consequences requiring prospective validation rather than established therapeutic effects.

## 2 Literature search and selection

A structured literature search was conducted in PubMed/MEDLINE, Embase, and Web of Science from database inception through September 6, 2026. Search concepts combined terms for PICS/CCI, critical illness, nutrition resistance or therapy, protein/amino acids/leucine, immunonutrition, omega-3 fatty acids, arginine, enteral nutrition/feeding patterns, circadian rhythm, metabolic dysfunction, and rehabilitation. English-language clinical guidelines, randomized trials, observational studies, reviews, and relevant mechanistic or translational studies were prioritized. Evidence was interpreted by directness to PICS (1): clinical studies in prospectively defined PICS; (2) clinical evidence from CCI or broader critical-care populations; and (3) mechanistic/translational evidence. Only the first was treated as direct PICS clinical evidence; the latter support extrapolation or biological plausibility, not PICS-specific efficacy. As a focused narrative synthesis, evidence certainty was not formally graded.

## 3 Guideline-based nutritional support and nutrition resistance in PICS

### 3.1 Guideline-based nutritional strategies in critical illness

Guidelines recommend early enteral nutrition, generally within 48 hours when oral intake is impossible and enteral feeding is feasible, while avoiding immediate full replacement during early acute illness (13, 14). Energy and protein delivery should progress with disease phase, metabolic tolerance, and stability rather than assuming uniformly beneficial higher early provision; requirements receive greater emphasis during stabilization and recovery (13, 19). Enteral nutrition may help preserve gastrointestinal integrity and gut-associated immune function, although such mechanisms do not establish consistent patient-centered benefit. Protein supplies substrates for maintenance and repair, but effects depend on disease phase, metabolic state, and anabolic responsiveness (20–23).

These principles remain foundational but derive from heterogeneous ICU populations, whereas PICS includes persistent inflammation, impaired immunity, metabolic dysregulation, and reduced anabolic responsiveness (3, 4). Recommended delivery may therefore fail to restore immune function, tissue regeneration, or anabolic balance (15–17). Nutritional adequacy and biological responsiveness are distinct; PICS support may need to address immune regulation, metabolic adaptation, and tissue repair alongside energy and protein requirements (12, 17).

### 3.2 Nutrition resistance in PICS: beyond nutrient delivery

Most nutrition evidence derives from heterogeneous acute ICU populations, whereas PICS emerges during prolonged critical illness/CCI (5, 16). Here, nutrition resistance is used as a working descriptor rather than a validated diagnosis: persistent catabolic or functional non-response despite apparently adequate and tolerated nutrient delivery after distinguishing underdelivery and feeding intolerance (5, 16). This definition is descriptive, not causal. No validated criteria, biomarker thresholds, or standardized test establish impaired utilization as the cause of non-response; assessment remains inferential and longitudinal (24). Nutrition resistance should not be inferred from persistent catabolism alone or used to justify a specific intervention; it remains a hypothesis-generating construct.

Persistent inflammation promotes proteolysis, amino acid oxidation, insulin resistance, and suppression of anabolic pathways (10, 25). With prolonged inactivity, these changes drive anabolic resistance, limiting muscle protein synthesis and recovery despite nutrient availability (25–27). Persistent immune activation associates with defective antigen presentation, lymphocyte dysfunction, and altered metabolism, compromising host defense and recovery (7, 9, 12). Thus, nutrient availability may be insufficient when nutrient sensing, metabolic adaptation, and substrate utilization remain impaired (12, 17).

Metabolic conditions may also modify nutritional responsiveness. Fluid/electrolyte disturbances, mitochondrial dysfunction, and impaired cellular energy metabolism occur during prolonged critical illness (11). ICU-acquired hypernatremia has been associated with greater inflammatory/catabolic features and longer stay in patients with PICS characteristics (28). These associations do not establish metabolic correction as PICS-specific nutritional therapy, but support considering the physiological environment alongside nutrient provision.

## 4 Pathophysiology-guided immunometabolic nutrition strategies

Viewing PICS as an immunometabolic disorder extends attention from nutrient provision to processes governing responsiveness and recovery (12, 29). Malnutrition may coexist and worsen muscle loss, whereas PICS additionally encompasses unresolved inflammation, immune dysfunction, anabolic resistance, metabolic disturbance, and defective repair (3, 6). These abnormalities may limit response despite adequate provision. Nutritional, rehabilitation, metabolic, and immune-modulating approaches are therefore complementary within this framework, not established PICS-specific therapies, and their relevance depends on phase, phenotype, and responsiveness.

### 4.1 Restoring anabolic responsiveness: protein, leucine, and rehabilitation-based strategies

Skeletal muscle wasting in PICS/CCI reflects inflammation, metabolic dysfunction, and impaired anabolic signaling (10, 25). IL-6/TNF-α promote proteolysis and reduced muscle protein synthesis (25), while inflammation and immobilization impair amino-acid responsiveness (27); simply increasing protein may therefore not restore anabolism. In EFFORT Protein, nutritionally high-risk mechanically ventilated adults were randomized within 96 h of ICU admission to higher (≥2.2 g/kg/day) versus usual (≤1.2 g/kg/day) protein targets. Higher dosing did not improve time to discharge alive and showed possible harm signals in acute kidney injury or greater organ failure (30). Because EFFORT studied acute critical illness rather than established PICS/CCI, it neither proves nutrition resistance nor defines later-recovery protein strategy.

Leucine supports protein synthesis and activates mTORC1 to stimulate muscle protein synthesis (31). In sepsis and chronic critical illness, inflammatory mediators and stress hormones may induce leucine resistance despite adequate leucine availability (32). Thus, overcoming anabolic resistance may require sufficient amino acids and metabolic conditions supporting anabolism.

Exercise and rehabilitation stimulate muscle protein turnover (26, 33, 34), and skeletal muscle can influence inflammation and immunity through myokines (35); inactivity promotes wasting and impaired metabolic adaptation (36). Timing and intensity remain uncertain. In TEAM, increased early active mobilization during mechanical ventilation did not improve days alive and out of hospital at 180 days and increased mobilization-related adverse events (37). TEAM was not PICS-specific and did not test rehabilitation during established PICS or later recovery, so it primarily cautions against assuming uniform benefit across phases.

Management should therefore prioritize anabolic responsiveness rather than simply increasing protein or leucine. Phase-appropriate nutrition and rehabilitation may provide complementary stimuli, but optimal dose, timing, and selection remain uncertain. PICS-specific studies should test whether inflammatory, immune, muscle, or anabolic-response markers identify patients most likely to benefit. Candidate studies should also distinguish preservation of muscle mass from recovery of strength and physical function, because these outcomes may respond differently to nutritional and rehabilitation interventions across the course of prolonged critical illness.

### 4.2 Targeting immune dysregulation and inflammatory resolution: phenotype-dependent roles of omega-3 fatty acids and arginine

Persistent inflammation and immune dysfunction in PICS may reflect impaired inflammatory resolution and immune recovery, not excessive inflammation alone. Immunomodulatory nutrition should follow immune phenotype, supporting resolution and immune competence rather than broadly suppressing immunity (3, 7). Omega-3 fatty acids and arginine are plausible approaches in PICS (5, 38), but their relevance is likely phenotype- and phase-dependent.

EPA and DHA may promote inflammatory resolution through specialized pro-resolving mediators, including resolvins, protectins, and maresins (39, 40), which regulate macrophage clearance, immune-cell trafficking, and tissue homeostasis (41, 42). However, this mechanism has not been shown to mediate clinical benefit in PICS. A 2026 meta-analysis of 29 randomized trials (2,551 critically ill adults) reported modest reductions in mechanical-ventilation duration and hospital stay, but populations and administration routes were heterogeneous (43). ESPEN 2023 permits fish-oil-containing parenteral lipid emulsions in selected patients but does not support routine high-dose omega-3-enriched enteral formulas (19). Thus, omega-3 remains biologically plausible but clinically unvalidated for PICS.

Arginine supports T-cell proliferation, macrophage function, and nitric oxide metabolism (44), while severe stress may increase requirements and depletion (44). Myeloid-derived suppressor-cell expansion is associated with persistent immunosuppression after severe sepsis (45), and increased arginase-1 can enhance L-arginine catabolism and impair T-cell responses (45, 46). This supports investigating arginine metabolism but does not validate MDSC or arginase markers for treatment selection. Supplementation also remains controversial in severe or uncontrolled sepsis because nitric oxide may worsen vasodilation and hemodynamic instability (47, 48). With limited direct PICS evidence, arginine-based immunonutrition should remain phenotype- and phase-dependent (48). Prospective studies are needed to determine whether these candidate immune markers predict treatment response before they can be used to guide supplementation in routine practice.

More broadly, emerging evidence from other disease contexts suggests that cellular processes linking metabolism, autophagic flux, and antigen presentation may influence immune competence (49), providing additional mechanistic directions for future PICS research. However, the relevance of these pathways to nutritional or immunomodulatory intervention in PICS remains unknown and requires direct investigation.

### 4.3 Nutrient timing and circadian-metabolic regulation during recovery

Nutrient timing may influence immunometabolic adaptation during recovery (50, 51). Continuous feeding remains appropriate when intolerance or instability limits alternatives (20), but sustained exposure may attenuate feeding–fasting signals related to autophagy, circadian regulation, and metabolic flexibility (51, 52). Because critical illness disrupts circadian/hormonal rhythms and mitochondrial function (53), intermittent or cyclic feeding may help restore rhythmicity during recovery, although direct PICS evidence is lacking (51, 54).

Sequential feeding combines continuous feeding during acute illness with intermittent feeding during recovery. One non-inferiority trial supported glycemic and gastrointestinal safety (55), while another reported higher albumin, total cholesterol, and lymphocyte counts than continuous feeding (56); these are nonspecific surrogates and neither trial was PICS-defined. In septic mice, sequential feeding preserved rhythmic hepatic circadian gene expression, whereas continuous feeding attenuated rhythmicity and increased steatosis (57). These findings support investigation, not PICS-specific efficacy.

Gut microbiota may also link timing to immunometabolic responses. Antibiotics, nutritional support, proton-pump inhibitors, and opioids contribute to ICU dysbiosis (58). A pilot study associated sequential feeding with less loss of bacterial diversity (59); a subsequent trial found no Shannon-diversity difference but increases in potentially beneficial taxa in disease-specific subgroups (56). These findings remain exploratory.

Sequential feeding is therefore a plausible phase-adapted approach, but evidence is limited, no prospectively defined PICS studies exist, and several reports come from the same group. Chrononutrition should remain an emerging mechanistic domain requiring independent validation and trials of meaningful outcomes and responsive phenotypes. Future studies should determine whether phase-adapted nutrient timing improves clinically meaningful outcomes and identify phenotypes most likely to benefit.

### 4.4 Metabolic milieu as a prerequisite for effective nutritional therapy

Nutritional effectiveness depends on both delivery and conditions governing utilization (13, 19). PICS may involve fluid/electrolyte disturbance, mitochondrial dysfunction, and impaired cellular energetics (11, 60). ICU-acquired hypernatremia has been associated with inflammatory/catabolic PICS features (28), and glycemic variability with higher mortality in septic patients with PICS (61). These associations identify metabolic instability but do not establish correction as PICS-specific therapy. Glycemic management should avoid hyperglycemia while minimizing hypoglycemia from intensive insulin therapy (62).

Accordingly, fluid/electrolyte management and individualized glycemic control are supportive prerequisites rather than stand-alone immunonutritional interventions (13, 19, 62). Meeting energy/protein targets may not ensure substrate utilization when cellular pathways remain impaired (63), and no specific nutritional intervention reverses mitochondrial abnormalities in PICS (11, 60). Metabolic stability should therefore be integrated with approaches to anabolic resistance, immune dysfunction, and circadian dysregulation without equating correction with modification of PICS itself; translation requires biomarker-based stratification and prospective PICS studies (6, 61). Serial assessment is therefore more informative than interpreting isolated metabolic abnormalities as direct therapeutic targets.

## 5 Discussion and future perspectives

This framework extends guideline-based support by addressing biological constraints on nutritional responsiveness (16, 64). Because PICS is heterogeneous and dynamic, with variable inflammation, immune dysfunction, anabolic resistance, and metabolic instability (3, 4, 6), nutritional and rehabilitation strategies should reflect disease phase, phenotype, and host responsiveness. **Table 1** summarizes the proposed strategies and uncertainties.

**Table 1:** Pathophysiology-guided nutritional strategies in PICS: biological targets, candidate phenotypes, and key uncertainties.

Intervention domain	Main biological barrier	Candidate phenotype/phase	Potential strategy	Current interpretation/key uncertainty
Anabolic responsiveness	Anabolic resistance and muscle wasting	Catabolic or anabolic-resistant phenotype; recovery phase	Adequate protein, leucine/EAA, phase-appropriate rehabilitation	Greater protein or rehabilitation intensity is not uniformly beneficial; optimal dose, timing, and patient selection remain uncertain.
Inflammatory resolution	Persistent inflammation and impaired resolution	Persistent inflammatory phenotype	Omega-3 fatty acids	Biologically plausible and phenotype-dependent; direct PICS-specific evidence remains limited.
Immune dysfunction	Persistent immunosuppression	Immune-suppressed phenotype; hemodynamically stable phase	Arginine-based immunonutrition	Potential benefit may depend on immune phenotype and disease phase; safety and efficacy require further study.
Circadian–metabolic regulation	Disrupted feeding–fasting rhythmicity and metabolic flexibility	Stabilization or recovery phase	Sequential, intermittent, or cyclic feeding	Preliminary mechanistic and clinical signals exist, but PICS-specific efficacy and external validation are lacking.
Metabolic milieu	Metabolic instability limiting nutrient utilization	Patients with glycemic, fluid, or electrolyte instability	Individualized glycemic control and fluid/electrolyte management	Evidence is primarily correlational; whether correction of metabolic instability beyond standard supportive care enhances nutritional responsiveness or improves PICS-specific outcomes remains unproven.

Strategies are presented as phenotype- and phase-dependent approaches that complement guideline-based nutritional support rather than established PICS-specific therapies. Supporting evidence is discussed in the corresponding sections of the main text.

### 5.1 Why responses to nutritional interventions may differ among patients

Energy and protein targets remain foundational (13, 14), but biological responsiveness varies in PICS (16, 64). Contributions from inflammation, immune suppression, anabolic resistance, and metabolic dysfunction vary among patients and over time (3, 4, 6). Anabolic benefit depends on preserved muscle responsiveness (25, 26, 65), while immunomodulatory effects may differ with inflammatory activation, impaired resolution, or immune suppression (47, 48, 66). Pathophysiology-guided nutrition should therefore identify modifiers of response rather than replace requirements, integrating disease phase, immune status, anabolic responsiveness, and metabolic characteristics (15, 64).

### 5.2 Major controversies in immunometabolic nutrition for PICS

Direct interventional evidence in prospectively defined PICS populations remains scarce (5, 6, 16, 38); most rationale comes from heterogeneous ICU populations, mechanistic studies, or other phenotypes.

Protein therapy illustrates the tension between substrate availability and responsiveness. Optimal PICS dose and timing remain uncertain (67); muscle protein synthesis can remain blunted despite amino-acid availability (65), with impaired mTORC1 signaling in experimental sepsis (32). EFFORT Protein found no benefit from higher delivery and possible harm in selected high-risk patients (30). Although not PICS-specific, these data support tailoring protein to phase, organ dysfunction, and anabolic responsiveness (68).

For immunonutrition, omega-3 fatty acids and arginine may affect inflammatory resolution and immune function (41, 48), but benefits are inconsistent and responsive populations uncertain (66). Because inflammation and immune suppression may coexist, future studies should prioritize phenotype, phase, and treatment–phenotype interactions rather than universal supplementation (47, 48).

For nutrient timing, intermittent or cyclic feeding may better preserve feeding–fasting and circadian-metabolic signaling during recovery (51), but evidence for patient-centered benefit, especially in PICS, remains limited (51, 52, 69). Sequential feeding is preliminary; optimal patterns may depend on trajectory, gastrointestinal tolerance, metabolic status, and recovery phase (20, 70).

### 5.3 Biological stratification and biomarker-guided nutrition

Translation requires identifying the dominant biological disturbance and whether it predicts response (6). The magnitude and trajectory of inflammation, immunosuppression, and catabolism vary among patients (4), while conventional assessment provides limited insight into determinants of responsiveness (15, 64).

Candidate stratification domains include inflammatory activity/resolution; immune-cell number and function (9, 45, 71); muscle mass, function, and anabolic responsiveness (25, 65); and glycemic variability (61). These features may define multidimensional phenotypes but are not established decision rules.

Future phenotyping may incorporate transcriptomic, proteomic, metabolomic, or other multi-omics signatures. In other diseases, multidimensional transcriptomic signatures can characterize immune microenvironments and predict treatment response, providing a methodological paradigm for PICS biomarker development (72). Such signatures require independent development and validation in critical illness/PICS before guiding treatment. The principal translational question is whether molecular signatures add predictive information beyond conventional inflammatory, immune, metabolic, and functional measures.

Prognostic biomarkers alone are insufficient because risk does not necessarily predict treatment response. Given evolving immune and metabolic phenotypes (4, 71), biomarker-guided nutrition should use longitudinal rather than single-time-point stratification and test biologically defined subgroups at specific disease phases (64, 73).

### 5.4 From mechanistic understanding to PICS-specific clinical translation

Clinical translation remains challenging (12, 64) because most intervention studies involve heterogeneous critically ill populations rather than prospectively defined PICS cohorts (5, 15). As PICS evolves from acute stress toward persistent immune dysfunction, anabolic resistance, metabolic impairment, and poor recovery (4), nutritional and rehabilitation strategies should be evaluated across disease trajectories rather than assumed to have uniform effects.

Management must also align with prognosis and goals of care. In advanced or poorly reversible illness, artificial nutrition should be weighed against likelihood of meaningful recovery, expected benefit, burden, patient preferences, and proportionality. Near the end of life, correcting nutritional or immunometabolic abnormalities should not become an isolated objective when artificial nutrition is unlikely to provide meaningful patient-centered benefit (74).

Future trials should prospectively define PICS, use longitudinal phenotyping, and test effect modification by phase and biological subgroup. Outcomes should extend beyond delivery targets and biochemical surrogates to muscle function, secondary infections, physical recovery, discharge disposition, quality of life, and long-term survival (75), while assessing biological target engagement where feasible.

Scalable longitudinal phenotyping may include serial body-composition assessment and integration of clinical, imaging, immune, and metabolic data. Automated imaging may objectively track skeletal muscle and other body-composition trajectories and help characterize catabolic/recovery phenotypes (76). Such multidimensional approaches remain research tools until prospectively validated against meaningful outcomes. Computational integration may further help identify biological trajectories or treatment-responsive subgroups, but methodological performance alone would not establish clinical utility.

Translation ultimately requires reproducible links among phenotype, intervention responsiveness, and clinical outcome. Until then, biomarker- and phenotype-guided nutrition should support hypothesis generation, patient stratification, and PICS-specific trial design rather than constitute standard care.

## 6 Conclusion

PICS combines persistent immunometabolic and catabolic dysfunction, while inadequate intake and malnutrition may further impair recovery. Guideline-based nutrition remains essential, but adequate delivery may not overcome impaired responsiveness from immune dysfunction, anabolic resistance, and metabolic disturbance. Management may integrate established energy/protein targets with disease phase, immunometabolic characteristics, and anabolic responsiveness. Pathophysiology-guided nutrition should complement, not replace, guideline-based support. This conceptual, hypothesis-generating framework requires prospective evaluation in well-defined PICS populations using longitudinal biological stratification and meaningful recovery outcomes. Prospective studies should prioritize clinically meaningful recovery outcomes rather than nutritional targets alone.
